# Targeted adaptive long-read sequencing for discovery of complex phased variants in inherited retinal disease patients

**DOI:** 10.1038/s41598-023-35791-4

**Published:** 2023-05-26

**Authors:** Kenji Nakamichi, Russell N. Van Gelder, Jennifer R. Chao, Debarshi Mustafi

**Affiliations:** 1 Department of Ophthalmology, Roger and Karalis Johnson Retina Center, University of Washington, Seattle, WA 98109 USA; 2 Brotman Baty Institute for Precision Medicine, Seattle, WA 98195 USA; 3 Division of Ophthalmology, Seattle Children’s Hospital, Seattle, WA 98105 USA

**Keywords:** Hereditary eye disease, Retinal diseases, Diagnosis, Genetic testing, Medical genetics, Sequencing

## Abstract

Inherited retinal degenerations (IRDs) are a heterogeneous group of predominantly monogenic disorders with over 300 causative genes identified. Short-read exome sequencing is commonly used to genotypically diagnose patients with clinical features of IRDs, however, in up to 30% of patients with autosomal recessive IRDs, one or no disease-causing variants are identified. Furthermore, chromosomal maps cannot be reconstructed for allelic variant discovery with short-reads. Long-read genome sequencing can provide complete coverage of disease loci and a targeted approach can focus sequencing bandwidth to a genomic region of interest to provide increased depth and haplotype reconstruction to uncover cases of missing heritability. We demonstrate that targeted adaptive long-read sequencing on the Oxford Nanopore Technologies (ONT) platform of the *USH2A* gene from three probands in a family with the most common cause of the syndromic IRD, Usher Syndrome, resulted in greater than 12-fold target gene sequencing enrichment on average. This focused depth of sequencing allowed for haplotype reconstruction and phased variant identification. We further show that variants obtained from the haplotype-aware genotyping pipeline can be heuristically ranked to focus on potential pathogenic candidates without a priori knowledge of the disease-causing variants. Moreover, consideration of the variants unique to targeted long-read sequencing that are not covered by short-read technology demonstrated higher precision and F1 scores for variant discovery by long-read sequencing. This work establishes that targeted adaptive long-read sequencing can generate targeted, chromosome-phased data sets for identification of coding and non-coding disease-causing alleles in IRDs and can be applicable to other Mendelian diseases.

## Introduction

Inherited retinal diseases (IRDs) affect 1 in 3000 individuals^1,2^ and are the leading cause of inherited blindness^3^. IRDs are genetically heterogeneous disorders with more than 300 causative genes identified^4^. Retinal disease can be one of the first presenting features of a syndromic condition^3^. Early genetic diagnosis of such conditions minimizes potential extraocular morbidity^5^. Currently, short-read exome-based sequencing panels are the most commonly used approach to genetically diagnose IRD patients^6^. However, chromosomal maps for allelic variant discovery cannot be optimally reconstructed from short-reads^7^. Patients with complex structural variants and pathogenic non-exomic, or non-coding, variants can be missed by such approaches. This creates diagnostic dilemmas in AR IRDs, the most prevalent inheritance pattern of IRDs^8^. Up to 20–30% of AR IRD patients have one or no identified disease-causing variants from short-read exome sequencing^9,10^. These cases of missing heritability can be attributed to variants in non-coding regions of the genome^11–13^, which can be better elucidated by genome sequencing^14^.

Long-read genome sequencing strategies offer an approach for discovery of complex phased variants^7^. The ONT long-read sequencing platform provides the unique benefit of data acquisition flexibility with real-time analysis that allows target enrichment by directly rejecting or accepting DNA molecules during sequencing without specialized sample preparation^15–17^. The ability to sequence native DNA and RNA molecules also eliminates amplification bias while preserving base modifications^18–20^. Since sequences of biological interest often comprise only a small fraction of the human genome, this bioinformatic method avoids wasting sequencing bandwidth on uninformative reads to allow increased depth of coverage from the same sequencing effort. Adoption of this technique, however, has been limited, as previous work has suggested that accuracy per read is lower for ONT long-reads compared to Illumina short-read sequencing^21^.

In this work we applied targeted, adaptive long-read sequencing to study the most prevalent syndromic IRD^22^, Usher syndrome type 2 (USH2)^23^. USH2 is an AR ciliopathy characterized by progressive retinal degeneration and sensorineural hearing loss^24,25^. USH2 is most commonly caused by variants in *USH2A*^26^, which is composed of 72 exons spread over ~ 800,000 base pairs^27^. Whereas this is a large gene, in total it constitutes less than 0.1% of total DNA in the human genome^28^. This means greater than 99% of sequences analyzed by whole genome sequencing approaches are not informative. Targeted enrichment avoided wasting sequencing bandwidth on uninformative reads to allow much deeper coverage from the same sequencing effort. We demonstrate that the adaptive sequencing methodology on the ONT platform from blood-derived genomic DNA of a family with USH2 allowed us to focus our sequencing depth to the *USH2A* locus to achieve greater than 12-fold relative enrichment on average. Moreover, the long-read lengths allowed us to resolve haplotype architecture for phased variant calling^29,30^. Furthermore, use of a haplotype-aware genotyping pipeline^31,32^ with heuristic ranking of the deleteriousness of identified variants narrowed down potential disease-causing variants sufficient to make a diagnosis based on the proband alone. More importantly, we show that use of a variant context matching method developed in this work to unravel discordant variants between short- and long-read alignments revealed that the concordance rates between short-read and long-read data exceed 96% for single nucleotide variants (SNVs). Finally, this work establishes that phased clinically relevant variants can be identified with higher precision and F1 scores from targeted long-read sequencing despite lower mean coverage compared to short-read genome sequencing, providing a rapid and clinically relevant method of disease variant discovery for definitive genotypic diagnosis.

## Results

### Targeted long-read sequencing enables selective enrichment of the USH2A locus

**Figure 1:**
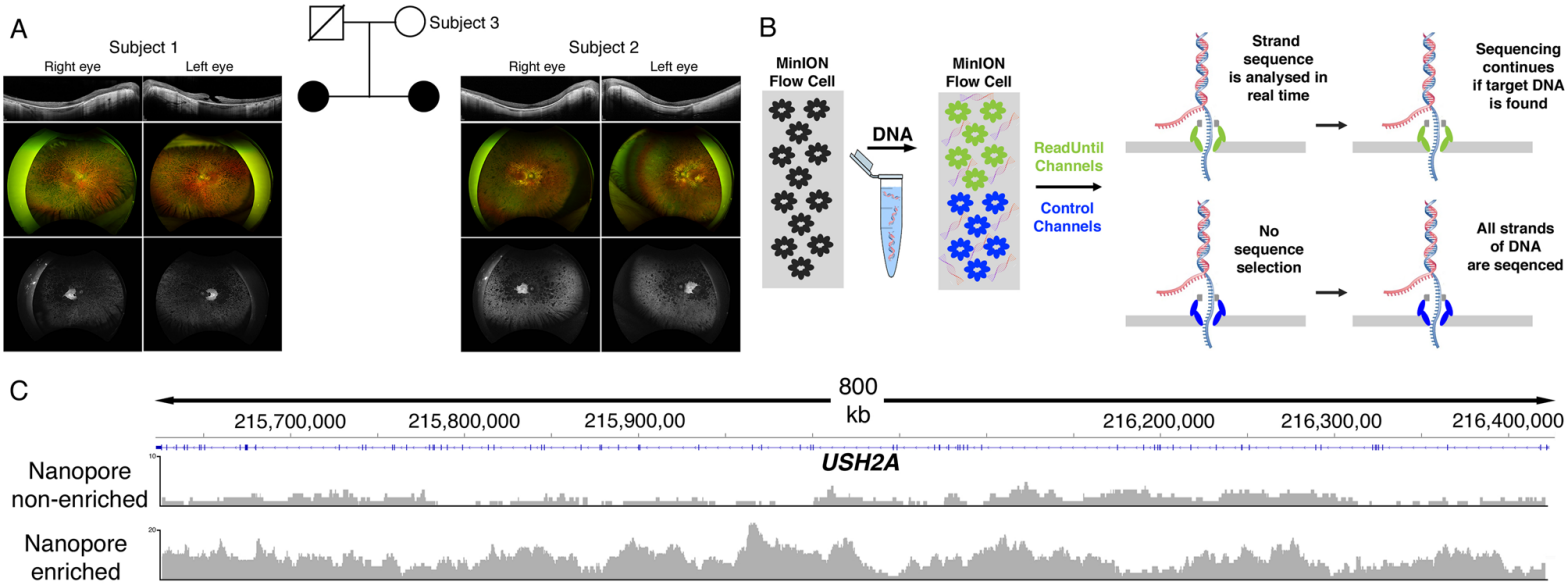
(**A**) Genogram of the subjects enrolled in the study. Subjects 1 and 2 had clinical features of Usher Syndrome as demonstrated by findings on optical coherence tomography, color fundus and fundus autofluorescence imaging of both eyes. (**B**) To demonstrate selective enrichment of the *USH2A* locus, adaptive sampling (‘ReadUntil’) was implemented on half of the channels (green) with the remaining half channels (blue) acting as control. By comparing the ratio of reads from the green and blue channels of the region of interest, the fold enrichment can be calculated. (**C**) Adaptive sampling led to a greater than 12-fold increase on average in sequencing coverage of the *USH2A* locus from human blood compared to the control, non-enriched channels, as evidenced by the coverage depth plots Haplotyping was not possible using non-enriched sequencing but accomplished with enriched sequence obtained from adaptive sampling.

For this study, we enrolled two siblings (Subjects 1 and 2) with clinical features consistent with USH2 along with their unaffected mother (Subject 3) (Fig. 1A). Their father was deceased but had no prior clinical history suggestive of Usher Syndrome. Short-read exome panel testing of Subject 1 had revealed two pathogenic variants in the *USH2A* gene. No phase information was available to verify that the two variants resided in *trans*. No other family member had undergone genetic testing. To focus sequencing effort on the *USH2A* gene locus, we successfully applied targeted long-read sequencing using adaptive sampling^15–17^ for the ONT platform to determine the phase of the two identified variants in Subject 1 and carry out sequencing for the first time in Subjects 2 and 3.

**Figure 2:**
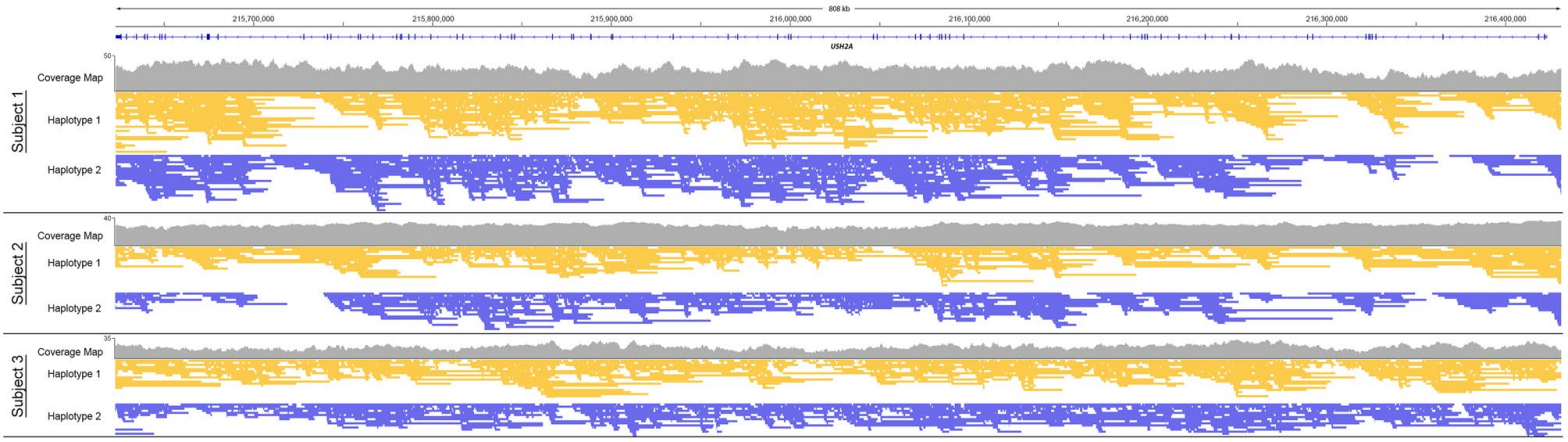
Coverage maps and read coverage of the targeted *USH2A* gene locus of the subjects reveal 100% base coverage at mean sequencing depths of 33×, 19× and 17×, respectively. The depth of sequencing allowed for assembly into haploid genomes (Haplotype 1 in yellow and Haplotype 2 in purple).

Adaptive sampling was utilized on the ONT platform by rapidly resetting pores processing sequences that were not contained in the *USH2A* locus^15^ (Fig. 1B). After a few hundred bases of a DNA strand are basecalled, the sequence is compared to the reference sequence of the USH2A locus and if there is no match, then the strand is ejected to allow for another strand to be assayed. Deliberately rejected reads had an N_50_ (the read length such that reads of this length or greater sum to at least half the total bases) of only 406 bases compared with adaptive N_50_ of 9499 bases, illustrating how this technology allows one to quickly reject a read and move to sample another piece of DNA. Overall, targeted adaptive sequencing resulted in greater than 12-fold relative enrichment of the *USH2A* locus compared to non-adaptive sequencing, on average, and provided complete base coverage of *USH2A* gene locus (Fig. 1C). Focusing the sequencing bandwidth of the entire ONT flow cell targeting the *USH2A* locus resulted in mean sequencing depth of 33X for Subject 1, 19X for Subject 2, and 17X for Subject 3, which was sufficient to carry out haplotyping (Fig. 2).

### Phasing of clinically identified variants, variant annotation, filtration and curation of long-read data

**Figure 3:**
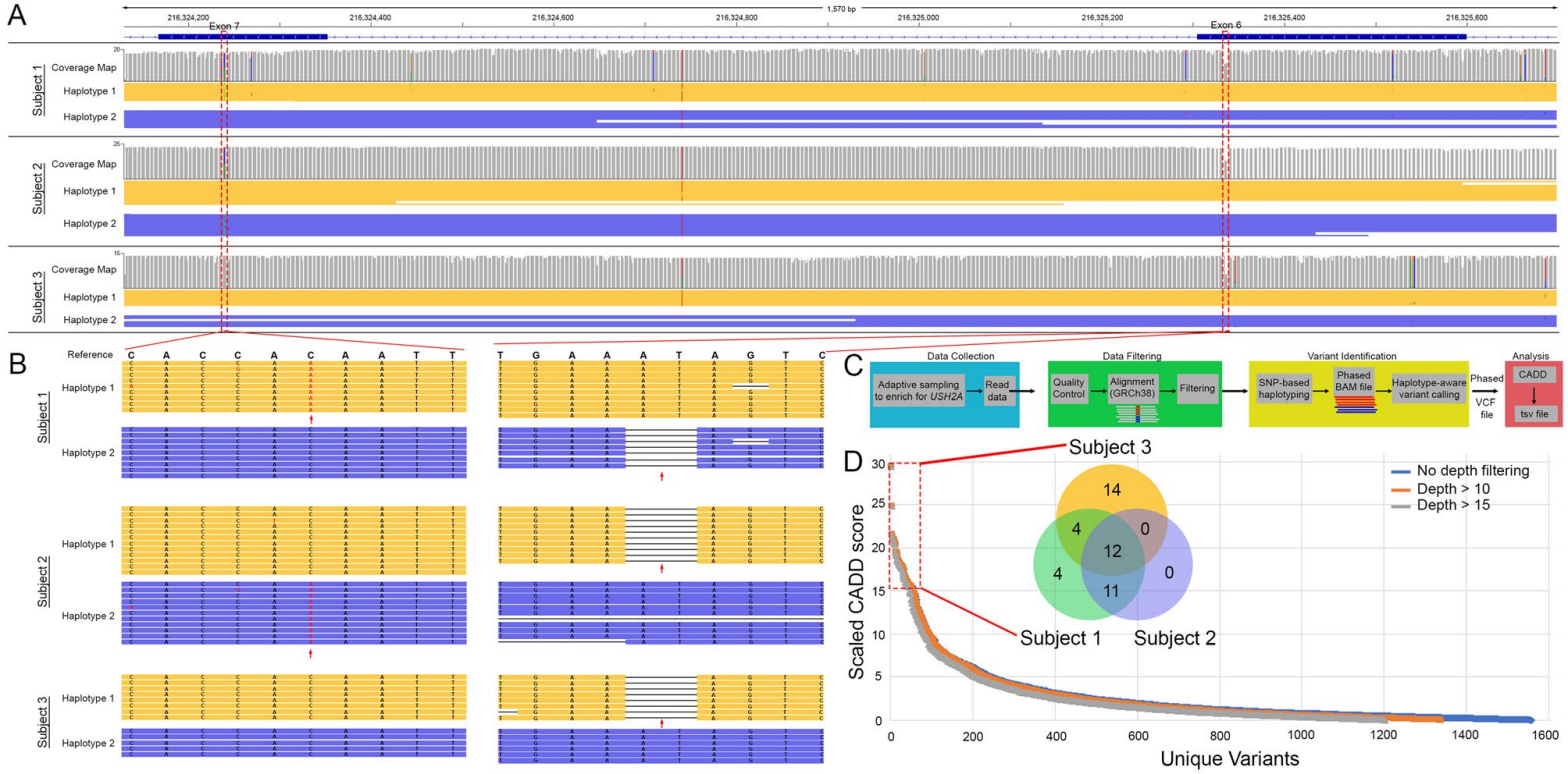
(**A**) Examination of the coverage maps of exons 6 and 7 and the intervening intronic region of *USH2A* shows the depth of coverage and reveals complete base coverage of reads mapping to haplotype 1 (yellow) and haplotype 2 (purple) for all three subjects. The red dashed boxes highlight the 2 regions where the identified pathogenic variants reside. (**B**) In exon 7 there is a SNV (C > A indicated by a red arrow) that is present on one haplotype in subjects 1 and 2 but absent in the unaffected subject 3. In exon 6, the red arrow shows the 2 base pair deletion that is present on one haplotype across all three subjects. (**C**) Schematic of our pipeline for long-read data collection, filtering, variant identification and analysis of the phased VCF files to generate a ranked list of deleterious variants. (**D**) Unique variants as a function of sequencing depth and scaled CADD scores are plotted to show that when using a cutoff of 15 for the CADD score one can narrow down to 45 with 23 potentially pathogenic causing variants shared between the diseased Subjects 1 and 2. Further investigation of the haplotype data can narrow that number down to 11 knowing that disease manifests in an AR inheritance pattern.

Examination of the haplotype data from Subject 1 revealed the two identified variants in *USH2A* from prior clinical sequencing resided in *trans*: a missense variant in exon 7 (c.1256G > T, p.Cys419Phe) and a deletion that resulted in a frameshift and early termination codon in exon 6 (c.1111_1112del, p.Ile371Phefs*3) (Fig. 3A, B). The same two variants were found to be in *trans* in Subject 2. Only the variant in exon 6 was detected in Subject 3, confirming her carrier status (Fig. 3B). Whereas these two variants were known from prior clinical sequencing of Subject 1, we found that implementation of data collection, filtering, haplotype-aware genotyping pipeline, and heuristic ranking of the identified variants by their deleterious nature from their scaled Combined Annotation Dependent Depletion (CADD) score^33,34^(Fig. 3C) ranked c.1256G > T and c.1111_1112del as the two most deleterious variants in the diseased subjects. To discover potentially causal variants we applied a cutoff on deleteriousness at a CADD score of 15, which reduced the search space to 45 variants across all 3 subjects (Fig. 3D). Examination of those variants present in both affected subjects further narrowed the search space to 23 variants. This was further reduced to 11 variants by comparing the haplotype data knowing that the variants must be in *trans* to manifest AR disease. Closer study of just Subject 1 revealed 31 deleterious ranked variants, but after comparing the haplotype data, we were able to reduce that to 14 variants. Thus, in the absence of familial data, the haplotype data can simplify the combinatorics to reduce the number of variants to a handful for disease discovery in the proband alone. Targeted long-read sequencing on the ONT system provided phased variants in USH2 patients, which in combination with our approach, demonstrated that methods to rank variants obtained from the haplotype-aware genotyping pipeline can focus on potential pathogenic candidates without a priori knowledge of the disease-causing variants.

### Comparison of targeted long-read data with whole genome short-read data of the same subjects reveal higher precision for variant discovery from long-read data

**Figure 4:**
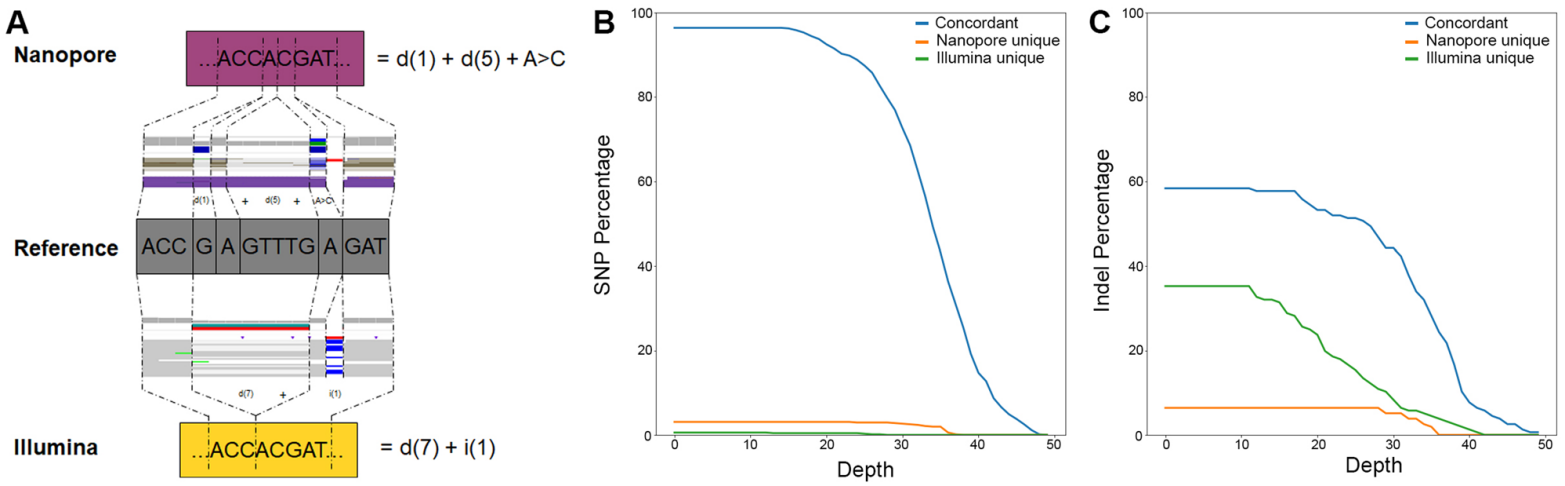
(**A**) Examination of the variants that were distinctly called between the two sequencing platforms revealed whereas the variants were different, they produced the identical output genomic sequence. In this example, the same sequence of ACCACGAT was generated by both sequencing techniques, but the long-read data assigned this variant due to a 1 base pair deletion, a 5 base pair deletion and a SNV (A > C) whereas the short-read data found the variant due to a 7 base pair deletion and 1 base pair insertion. The different cost functions for minimap2 (used for long-reads) and BWA-MEM (used for short-reads) led to the discordance. Fine tuning of the alignments taking these cases into account using a variant context matching method results in a concordance between long-read and short-read sequence calls of (**B**) over 96% for SNPs and (**C**) 58% for INDELs.

**Figure 5:**
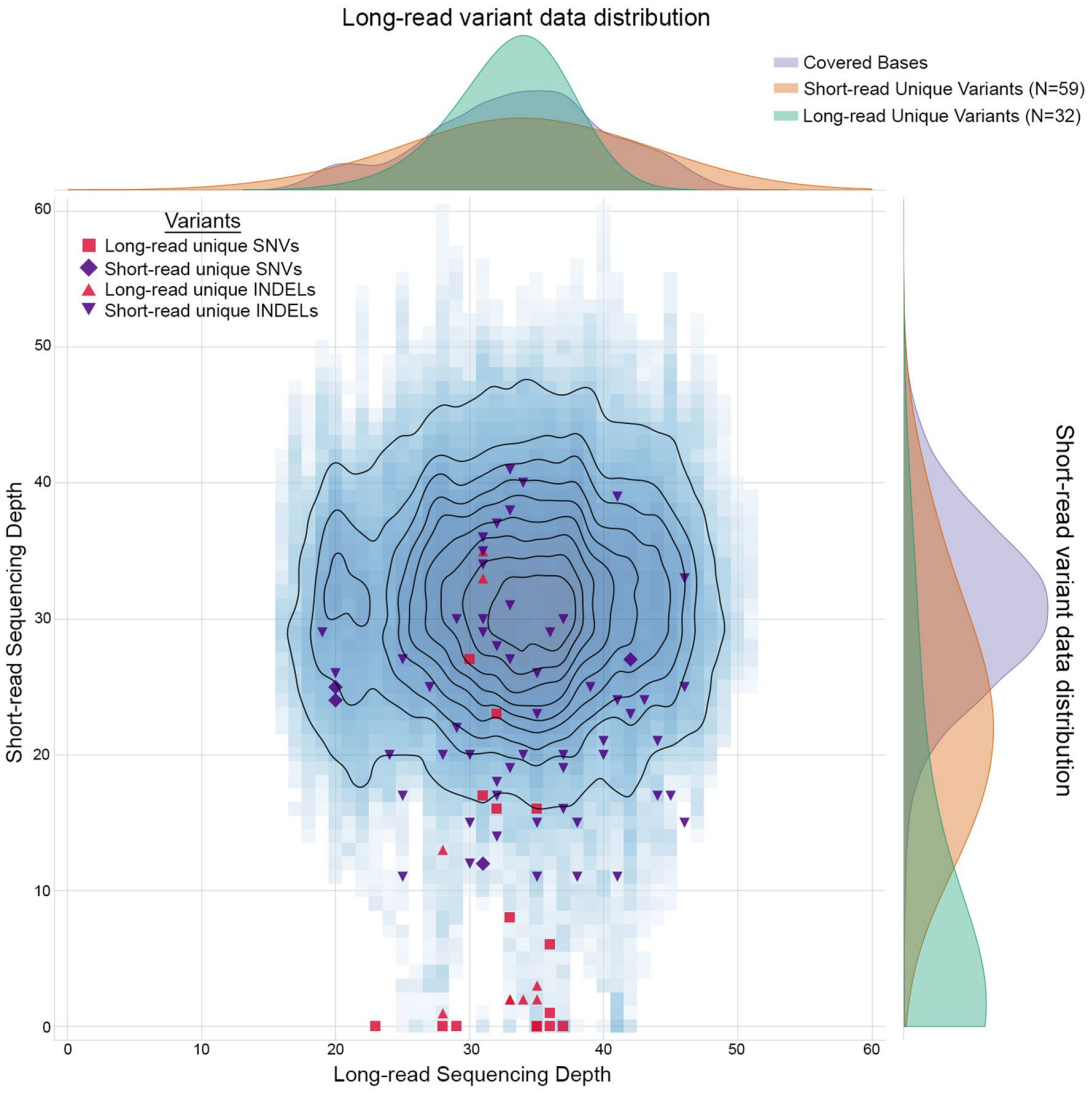
A kernel density estimation of the paired depth of sequencing from short- and long-read data was used to separate variant calls into quantiles (contoured edges) with the highest confidence quantile in the center. The variants (SNVs and INDELs) unique to each sequencing modality are highlighted where they fall in relation to the quantiles. The plots to the top and right of the depth plot illustrate the depth distribution of bases called by long- and short-read sequencing, respectively. Long-read sequencing provided coverage of 800,557 bases compared to 799,736 bases by short-read sequencing of the *USH2A* locus. The colored curves demonstrate the unique variants to each sequencing modality as a function of sequencing depth. Whereas the covered bases by each sequencing modality has a normal distribution, there is significant bias when sampling the short-read data. For the long-read data the peaks of the bases covered, long-read unique variants and short-read unique variants are closely centered around sequencing depth of 34. However, for the short-read data the peaks of the bases covered, long-read unique variants and short-read unique variants are at sequencing depths of 31, 21, and 1, respectively, demonstrating a shift to lower read depth for those unique in short-read data whereas the ones identified by long-read data are generally not covered by short-read sequencing.

We also carried out short-read sequencing of the three subjects, which generated a mean sequencing depth of 31×, 36×, and 33× for Subjects 1, 2, and 3, respectively. Our initial analysis revealed over 94% concordance of SNVs that agreed by location and phase called between subjects, which is higher than previously reported rates^35^. We focused further analysis on data from Subject 1 for whom comparable sequencing depth from both short-read (31×) and long-read (33×) experiments had been obtained. A closer examination of the variants that differed between the two platforms showed the underlying sequence was identical, but the alignments to the reference sequence differed (Fig. 4A). Applying a variant context matching tool to properly classify these perceived different calls resulted in a concordance of greater than 96% for SNVs (Fig. 4B) and 58% for indels (Fig. 4C) between the two sequencing modalities. We found that long-read sequencing provided greater base coverage of the *USH2A* gene locus, and more importantly, of the variants that were unique to the long-read data (22 SNVs, 10 indels) (Supplementary Table S1), the majority of them were not called by short-reads due to low coverage in those regions (17 SNVs, 7 indels) (Supplementary Fig. S1). Furthermore, no location of a variant was covered at a depth lower than 17 with long-read technology, whereas there was a significant proportion of genomic regions in which variants resided that fell below that threshold with short-read technology. In the context of variant discovery we evaluated all the discordant calls to determine why variants were excluded from a sequencing modality. A paired coverage plot showed that there was a large bias of a subset of variants found by long-reads that had poor coverage and quality by short-reads, however for those identified by short-reads no such trend was seen in the long-read data (Fig. 5). If we account for the high quality discordant variants that were unique to one technology then long-read technology demonstrates higher modified precision and F1 scores (Table 1).

**Table 1:** Precision, recall, and F1 metrics for SNVs and INDELs identified by long-read and short-read sequencing.

	Precision score	Recall score	F1 score
All variants long-read	0.9901	0.9313	0.9598
All variants short-read	0.9293	0.9604	0.9446
SNVs long-read	0.9930	0.9944	0.9937
SNVs short-read	0.9942	0.9928	0.9935
INDELs long-read	0.9691	0.6309	0.7642
INDELs short-read	0.6127	0.9667	0.7500

When accounting for high confidence discordant variants unique to a single sequencing modality, targeted long-read sequencing achieved higher F1 scores for all variants as well as for subsets of SNVs and INDELs. Of the discordant INDELs, short-read more accurately identifies INDELs, whereas long-read correctly flags them for discovery in subsequent analysis.

## Discussion

This work demonstrates the clinical utility of targeted long-read sequencing for variant detection and haplotyping in IRD patients. Long-read sequencing allows a more rapid turnaround time of a few days compared to traditional short-read approaches of 1–2 weeks necessary for library construction, sequencing, and data processing. Moreover, the depth of coverage of 10- to 15-fold required for haplotyping in AR diseases^36^ can be achieved optimally with just one ONT flow cell. Our data demonstrated mean enrichment of 12.4× per run with a single flow cell providing greater than 15× coverage. Furthermore, long-read targeted sequencing not only confirmed the clinically identified pathogenic variants in Subject 1, but that the variants, which reside 1095 bases apart, could be phased in the absence of trio testing to make a diagnosis from the proband alone. In detecting compound heterozygous variants, phasing information is invaluable since one of the variants might be de novo and data from parents are not always available to exclude the possibility that variants are in *cis*. Moreover, utilizing established deep-learning based approaches to heuristically rank identified variants, we demonstrate that variants can be prioritized without a priori knowledge of the pathogenic variants. The ability to phase long-read data allows further narrowing of discovered variants in cases of AR disease. More importantly, we address the discordance of variants identified by short-read and long-read technology by examining the perceived discordant variants. We found that the underlying genomic sequence can be identical but alignments differ between sequencing platforms, which we refined using variant context matching in this work to highlight a higher concordance rate of variant calls between sequencing modalities than previously reported. A rigorous estimation of the paired depth of sequencing elucidated that the precision and recall rates are superior in using targeted long-read data of the same subjects for variant discovery. Therefore, at the more modest read depths generated from long-read data compared to short-read data, we are able to identify clinically significant variants in *USH2A* and provide phase information to make a molecular diagnosis.

The ability to enrich for a large and complex genomic locus such as *USH2A* will be integral in explaining the missing heritability from non-coding variants, as they are difficult to classify and thus remain under-diagnosed^37^. The ability to enrich sequence for any target region makes this method adaptable to various IRDs, and is generalizable to any Mendelian genetic disorder. By sequencing the first 400–500 base pairs of a DNA molecule, adaptive sampling software can identify if the sequence contains the target region, otherwise the DNA strand is ejected from the pore. Our results confirmed that none of our rejected reads were in the targeted region *USH2A*. Moreover, this approach can be expanded from targeting a single genomic locus to multiple genomic loci^16^, encompassing genomic loci currently covered by the most comprehensive exome based clinical IRD panels.

To solve cases of missing heritability in IRDs following exome sequencing, a patient centered approach that focuses on non-coding and regulatory regions in disease genes of interest will be critical. This is especially important in the current era of genome editing technology, where identification of disease-causing variants is central to enrollment in possible treatment trials. Genetic diagnosis of patients with previously undiagnosed disease using next generation sequencing techniques can affect medical care in a variety of ways^38^. Bioinformatic pipelines for variant calling analysis of genome sequencing data must be precise and efficient for their integration into clinical diagnostics. We demonstrate that targeted adaptive long-read sequencing, which allows focused analysis of sequences of biologic interest, produces a phased variant set. The precision, recall and F1 scores with targeted long-read sequencing demonstrates the effectiveness of this approach for clinical interpretation in IRD patients. Moreover, the phased data sets allow clinical narrowing of relevant disease-causing variants and allows diagnosis from the proband alone. The sufficient accuracy of ONT adaptive sampling in identifying clinically relevant variants presents an evolution in addressing the missing heritability in retinal diseases and will be applicable to many other Mendelian disorders.

## Methods

### Ophthalmologic testing and genomic DNA extraction of the study patients

This study was approved by the institutional review board at the University of Washington (STUDY00014158). Written informed consent was obtained from all study subjects. Experiments were conducted according to the principles expressed in the Declaration of Helsinki. Clinical diagnosis of Usher Syndrome was based on history, ophthalmology, and audiology findings. Study participants underwent fundus color imaging (Optos), optical coherence tomography imaging (Spectralis HRA-OCT; Heidelberg Engineering), and Goldmann visual field perimetry. A venipuncture blood of 2 mL was obtained from study subjects and genomic DNA (gDNA) was isolated using the MagAttract High Molecular Weight genomic DNA isolation kit (Qiagen).

### Short-read library preparation and sequencing, variant calling, and variant annotation

Approximately 750 nanograms (ng) of gDNA was sheared using a Covaris LE220 focused ultrasonicator targeting 380 base pair inserts and then subjected to a series of library construction steps utilizing the Roche KAPA Hyper Prep kit (KR0961 v1.14) and NovaSeq 6000 S4 Reagent Kit v1.5 (300 cycles) for short-read Illumina sequencing. Base calls were generated in real-time on the Illumina NovaSeq6000 instrument. BAM files were aligned to a human reference (GRCh38) using Burrows-Wheeler Aligner; v0.7.15^39^. A Genome Analysis Toolkit (GATK)(28) (v4.2.6.1) based pipeline following the best practices was used^40,41^. In brief, the Illumina FASTQ files were aligned to the reference human genome GRCh38.p13 using the Burrows-Wheeler aligner BWA-MEM. Using SAMtools (1.13–5) the BAM file was then collated, the mate coordinate data appended, and duplicates marked. The reads were then filtered for pairing and minimum alignment quality score of MAPQ 50, then the supplementary, secondary, and optical duplicates were removed. The average base quality score for filtered reads that mapped to the targeted *USH2A* region was 29.3. The filtered BAM file was variant called using GATK HaplotypeCaller, and the output VCF underwent base quality score recalibration (BQSR) using GATK BaseRecalibrator, which used several large datasets of known genetic variation from the GATK resource bundle (1000G gold standard indels and high confidence SNVs, dbSNP138, Axiom Exome Plus, Hapmap3.3). The recalibration tables were then used with GATK ApplyBQSR to recalibrate the base quality scores, and the recalibrated BAM file then underwent a second round of variant calling using GATK HaplotypeCaller. The resulting VCF files underwent several variant quality score recalibration (VQSR) steps using GATK VariantRecalibrator in both SNV and INDEL modes using the respective reference variant datasets in the GATK resource bundle (dbSNP138, Omni2.5 1000G, 1000G high confidence SNVs, hapmap3.3 databases for the SNV recalibration; 1000G gold standard indels, Axiom Exome Plus, dbSNP138) and parameters tuned for whole genome sequencing. The 1000G gold standard indel database was used as a truth set for training the indel model, while the hapmap3.3, Omni2.5 1000G, and high confidence SNV databases were used as truth for the SNV model. The resulting recalibration table and tranches files were then applied using GATK ApplyVQSR sequentially in SNV and INDEL modes. The recalibrated VCF file was then split into SNVs and INDELs using GATK SelectVariants, and filtered using GATK VariantFiltration with tuned parameters. Notably, a score of MAPQ 50 was used for the RMS map quality. Bcftools (1.13–10) was used to concatenate, sort, and remove duplicates from the recalibrated VCF files. The VCF file was then split into passing variants with a minimum allele depth of 15.

### Long-read library preparation and long-read targeted enrichment of USH2A

For long-read library preparation, approximately 1200 ng of gDNA was used to make sequencing libraries using the ONT Ligation Sequencing Kit (SQK-LSK110) with slight modifications of the manufacturer’s protocol. As a modification to these instructions, 1.5× the suggested amount of AMPure XP beads were used and 80% (instead of 70%) ethanol was used for the bead washing steps. During the adapter ligation and clean up step, the Long Fragment Buffer was used to enrich for DNA fragments greater than 3 kilobases in length. Approximately 5–50 femtomoles of DNA were loaded onto an R9.4.1 flow-cell running on a Oxford Nanopore MinION Mk1B device.

A GPU accelerated version of guppy (v6.0.7; API version 10.1.0) was used for basecalling in real-time on two NVIDIA RTX A6000 GPUs using the “super-accurate” model parameters. Target regions of *USH2A* were enriched using Readfish^16^ adaptive sampling technology implemented during real-time sequencing. To perform adaptive sampling for in silico enrichment, we prepared the following FASTA file: chr1: 214622891–217423448. A 1 megabase buffer on each side of the USH2A gene was chosen to capture a genomic region the size of the gene on either side, but the size of the flanking region can be tailored to a smaller area by the experimenter depending on their interest on how much of gene flanking sequencing to enrich for. The adaptive sampling mode was set to enrich the *USH2A* locus on either 256 of the 512 available channels in each run or on all 512 channels. Reads were mapped using Minimap2 (v2.22-r1101). Sequencing experiments were run for up to 48–72 h with a nuclease flush and library reload after 24–48 h to recover maximal pores for continued sequencing.

### Sequence haplotagging, variant calling, and variant annotation of long-read data

FASTQ files were generated using Guppy and aligned to GRCh38 using minimap2^42^. The BAM file was collated, duplicates marked and the reads filtered for a minimum alignment quality score of MAPQ 50 and secondary, supplementary, and optical duplicates were removed using SAMtools. The average base quality score for filtered reads in the targeted *USH2A* region was 23. Variants were called using PEPPER and haplotyping was achieved using Margin. The DeepVariant pipeline was used to generate a phased variant call file (VCF)^31^. The Combined Annotation-Dependent Depletion (CADD) score, which integrates diverse genome annotations and scores any possible human SNV or indel event for their deleterious nature, was generated for each phased VCF, to provide a quantitative predictive of deleteriousness, pathogenicity, and molecular functionality of the identified variants. Discordant reads were examined using Python scripts available for download and use from GitHub (https://github.com/mustafilab).

## Supplementary Information

Supplementary Information.

## Data Availability

The gnomAD and additional data tools (i.e. CADD) used in this work are publicly accessible. The variant data in this study are included within the published article. Genome sequencing data are not publicly available due to privacy and patient anonymity issues. Genome sequencing data can be accessed upon reasonable request to the corresponding author.
