# Supplementary material for: Targeted adaptive long-read sequencing for discovery of complex phased variants in inherited retinal disease patients
Source: Sci Rep. 2023 May 26;13:8535. doi: 10.1038/s41598-023-35791-4 (PMC10219926; doi:10.1038/s41598-023-35791-4)
Supplement: Supplementary file 1 — Supplementary Information. [file 41598_2023_35791_MOESM1_ESM.pdf]

Supplementary Material

Targeted adaptive long-read sequencing for discovery of complex phased variants in inherited retinal disease patients

Kenji Nakamichi[1], Russell N. Van Gelder[1], Jennifer R. Chao[1], Debarshi Mustafi[1,2,3*]

[1]Department of Ophthalmology and Roger and Karalis Johnson Retina Center, University of Washington, Seattle, WA, 98109, [2]Brotman Baty Institute for Precision Medicine, Seattle, WA 98195, [3]Division of Ophthalmology, Seattle Children's Hospital, Seattle, WA, 98105

***Corresponding Author:** Department of Ophthalmology and Roger and Karalis Johnson Retina Center, University of Washington, Seattle, WA, 98109, USA; Telephone: 206-616-9305; Fax: 206-897-4320; E-mail: debarshi@uw.edu

Supplementary Table S1. Unique *USH2A* variants called by targeted long-read sequencing. The locations along with reference and alternate sequences of the 10 INDELs and 22 SNVs unique called by long-read sequencing are shown. The majority of variants (24/32) are not called by short-read sequencing due to low read coverage in those regions.

Location (chromosome 1)	Reference	Alternate	variant_type	Low coverage on Illumina
215627449	C	T	SNV	NO
215627453	C	T	SNV	NO
215630480	GTGTATATATATATA	G	INDEL	NO
215733213	G	A	SNV	NO
215743422	A	ATG	INDEL	YES
215743424	A	G	SNV	YES
215743426	A	G	SNV	YES
215743429	G	T	SNV	YES
215743431	C	T	SNV	YES
215743433	C	T	SNV	YES
215743435	C	T	SNV	YES
215743440	A	G	SNV	YES
215743442	A	G	SNV	YES
215743444	A	G	SNV	YES
215743447	A	T	SNV	YES
215743451	A	T	SNV	YES
215743461	G	C	SNV	YES
215747980	C	T	SNV	YES
215835210	T	G	SNV	NO
215836391	A	C	SNV	YES
215836477	T	TA	INDEL	YES
215836480	A	AT	INDEL	YES
215836498	A	ATATAATAT	INDEL	YES
215836526	T	TA	INDEL	YES
215836528	A	AT	INDEL	YES
215836553	A	AT	INDEL	YES
215912515	G	A	SNV	YES

215952013	C	T	SNV	YES
215952040	C	A	SNV	YES
216074304	A	C	SNV	NO
216076522	AT	A	INDEL	NO
216141954	GA	G	INDEL	NO

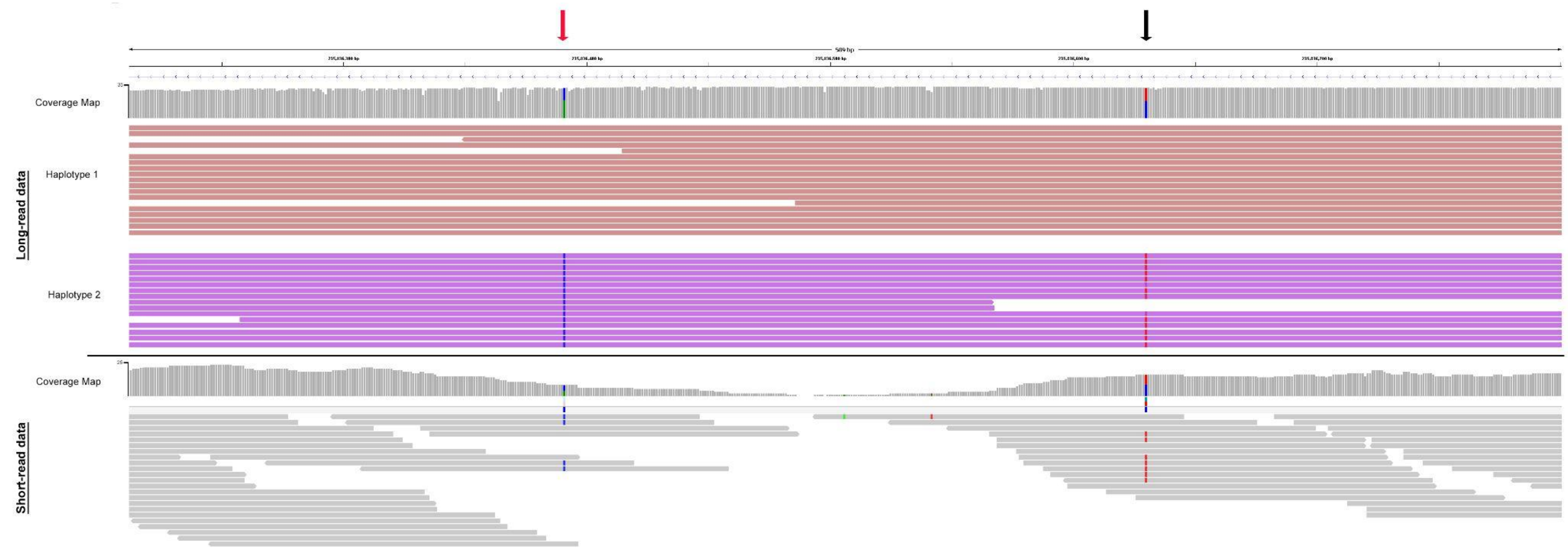

Supplementary Figure S1. Coverage map and read coverage of a genomic region encompassing intron 47 of the *USH2A* gene of long-read and short-read data of Subject 1. Compared to the long-read data that has complete coverage of this entire region, there are regions that are poorly covered by the short-read data. Both technologies are able to identify a variant denoted by the black arrow at the top. In the long-read data one can conclude it is a clear heterozygous variant as evidenced by its presence only on Haplotype 2 of the data. A SNV at 215836391-A-C, a common variant found in gnomAD with allelic frequency of 0.0364, is denoted by the red arrow at the top and is uniquely identified by the long-read data only. The variant is clearly seen to segregate on Haplotype 2 of the data. There are a few reads in the short-read data where the SNV is identified but because of their low quality scores, the variant is not reliably called by this technology.
